# A case–control study in Shanghai of fruit and vegetable intake and endometrial cancer

**DOI:** 10.1038/sj.bjc.6602609

**Published:** 2005-05-10

**Authors:** M H Tao, W H Xu, W Zheng, Y T Gao, Z X Ruan, J R Cheng, Y B Xiang, X O Shu

**Affiliations:** 1 Center for Health Services Research, Department of Medicine, Vanderbilt-Ingram Cancer Center, Vanderbilt University School of Medicine, 6009 Medical Center East, 1215 21st Avenue South, Nashville, TN 37232-8300, USA; 2 Currently a doctoral student at the Department of Epidemiology, School of Public Health, University of California at Los Angeles, Box 951772, Los Angeles, CA 90095-1772, USA; 3 Department of Epidemiology, Shanghai Cancer Institute, #25 2200 Xie Tu Road, Shanghai 200032, People's Republic of China

**Keywords:** vegetables, fruit, endometrial cancer

## Abstract

In a population-based case–control study of 832 incident endometrial cancer cases and 846 frequency-matched controls among Chinese women in Shanghai, using a validated food-frequency questionnaire, dietary habits were estimated by in-person interviews. Total vegetable consumption was inversely associated with endometrial cancer risk (highest quartile *vs* lowest: OR=0.69, 95% CI 0.50–0.96). The risk was reduced with increasing intake of dark green/dark yellow vegetables (trend test, *P*=0.02), fresh legumes (trend test, *P*<0.01), and allium vegetables (trend test, *P*=0.04). Fruit consumption was unrelated to risk. These results suggest that high consumption of certain vegetables may reduce the risk of endometrial cancer.

The incidence rates of endometrial cancer are highest in the United States and Canada and lowest in Asia and Africa, while Asian women who have migrated to the United States have an intermediate incidence (Parkin *et al*, 2003). Such international variation suggests that lifestyle factors, including dietary consumption patterns may play a major role in the aetiology of this disease (Purdie and Green, 2001). Fruits and vegetables are high in dietary fiber, which may influence the metabolism, recirculation, and excretion of oestrogen. They also contain numerous bioactive components, including *β*-carotenes, isothiocyanates, phyto-oestrogens, folic acid, and vitamins C and E which have been postulated to reduce the risk of many cancers (Rao, 1996; Riboli and Norat, 2003; Key *et al*, 2004). However, studies on the relationship between fruit and vegetable consumption and endometrial cancer risk have been limited and the results inconsistent (La Vecchia *et al*, 1986; Barbone *et al*, 1993; Levi *et al*, 1993; Shu *et al*, 1993; Zheng *et al*, 1995; Goodman *et al*, 1997a, 1997b; McCann *et al*, 2000; Littman *et al*, 2001; Petridou *et al*, 2002; Terry *et al*, 2002).

We evaluated the association of fruit and vegetable intake levels with endometrial cancer in a population-based case–control study conducted in Shanghai, China, where the incidence of endometrial cancer is substantially lower than that in the United States and where consumption of vegetables, especially leafy green vegetables, is high.

## MATERIALS AND METHODS

The Shanghai Endometrial Cancer Study is a population-based case–control study. Cases consisted of permanent female residents of urban Shanghai, who were newly diagnosed with endometrial cancer between January 1997 and December 2001 and who were between 30 and 69 years of age. Through the population based Shanghai Cancer Registry, 982 eligible endometrial cancer cases were identified during the study period and in-person interviews were completed for 832 (84.7%) of them. The major reasons for nonparticipation were refusals (73 cases, 7.4%), death before interview (36 cases, 3.7%), inability to locate the subject (22 cases, 2.2%), absence during the study period (10 cases, 1.0%), and other miscellaneous reasons (9 cases, 0.9%).

The Shanghai Resident Registry, which registers all permanent residents of urban Shanghai, was used to randomly select female controls who were frequency-matched to cases by age (5-year intervals). The number of controls in each age-specific stratum was determined in advance according to the age distribution of incident endometrial cancer cases in 1996. Women who had had a hysterectomy were not eligible (*n*=36). Interviews were completed for 846 (72.6%) of the 1165 eligible controls. Reasons for nonparticipation of controls included refusal (277 controls, 23.8%) and absence during the study period (42 controls, 3.6%).

All study participants were interviewed in person by trained interviewers and were measured for weight, circumferences of the waist and hips, and sitting and standing heights. The standardised, structured questionnaire used for the study covered demographic factors, menstrual and reproductive history, hormone use, usual dietary habits, prior disease history, physical activity, tobacco and alcohol use, weight history, and family history of cancer. Usual dietary habits over the past 5 years were assessed through in-person interviews using a validated, quantitative, food-frequency questionnaire (FFQ), which was designed to capture more than 85% of foods consumed in Shanghai. The FFQ listed 71 food items, including 30 fresh vegetable items and eight fruit items. Two measurements were taken with a tolerance of <1 cm for height and circumferences, and a tolerance of 1 kg for current weight. A third measurement was taken if the difference between the first two was larger than the tolerances stated above. The average of the two closest measurements were used to calculate body mass index (BMI) (kg m^−2^) and waist–hip ratio (WHR).

During the interview, each participant was first asked how frequently she consumed a specific food or group of foods (per day, week, month, year, or never), followed by a question on how many liangs (=50 g) were eaten per unit of time (day, week, month, or year) during the previous 5 years, ignoring any recent changes. For seasonal foods, each participant was asked to describe their consumption during the month(s) when the food was available. Average daily intakes were estimated by calculating the percentage of months that a food was on the market over a 1-year period.

Menopausal status was defined as no menstruation during the past 12 months, excluding those lapses caused by pregnancy or breastfeeding. For premenopausal women, years of menstruation were calculated by taking age at diagnosis (cases) or interview (controls) and subtracting age at menarche. For postmenopausal women, years of menstruation were calculated by taking age at menopause and subtracting age at menarche. For both groups, years with oral contraceptive use and years of pregnancy were subtracted from the total years of menstruation.

Statistical analyses were conducted using SAS Version 9.1 (SAS Institute, Cary, NC, USA). Quartile distributions among controls were used to categorise dietary intake variables. Odds ratios (OR) and corresponding 95% confidence intervals (95% CI) were used to estimate the association of endometrial cancer risk with intake of selected food groups. Unconditional logistic regression was used to obtain maximum likelihood estimates of ORs and their 95% CI, after adjusting for potential confounding variables. Age was included as a continuous variable throughout the data analyses, and categorical variables were treated as dummy variables in the model. Trend tests were conducted by treating categorical variables as the ordinal values in the models. Analyses stratified by menopausal status and BMI were conducted to examine whether the vegetable and endometrial cancer association was modified by the latter two factors. All statistical tests were based on two-sided probability and a significance level of *P*⩽0.05.

## RESULTS

Table 1 shows comparisons of cases and controls on demographic factors, known risk factors for endometrial cancer, total energy intake, and total meat and fish intake. Compared to controls, cases had an earlier age at menarche, later age at menopause, longer duration of menstruation, and consumed more meat and fish. Cases were more likely to have a higher educational level; a family history of colorectal, endometrial, or breast cancer among first-degree relatives; a history of diabetes; a higher BMI or WHR; a higher frequency of nulliparity; and lower frequency of using oral contraceptives (OC). All of the above variables were considered to be potential confounders and were adjusted for in subsequent analyses. There were no statistically significant differences between cases and controls regarding family income, hormone replacement therapy, adult height, physical activity, and usual intake of energy.

The multivariable-adjusted associations of fruit and vegetable consumption with risk of endometrial cancer among all subjects are presented in Table 2. An association of soyfood with endometrial cancer has been previously reported (Xu *et al*, 2004). Soyfood was, therefore, not included as an independent food group in the subsequent food group analyses. After adjusting for total energy intake, total meat and fish intake, BMI, and other potential confounders, increased consumption of total vegetables, dark green/dark yellow vegetables, fresh legumes, and allium vegetables were inversely associated with risk of endometrial cancer. The ORs and 95% CIs for the highest quartile *vs* the lowest quartile intake of these vegetables were 0.69 (0.50–0.96), 0.70 (0.52–0.95), 0.70 (0.52–0.96), and 0.76 (0.56–1.03), respectively. A significant dose–response relationship was observed for intake of total vegetables, dark green/dark yellow vegetables, fresh legumes, and allium vegetables.

Consumption of watermelon contributed 55% and 54% of fruit intake for cases and controls, respectively. As watermelon consumption is heavily influenced by seasonal supply and an accurate measurement of consumption level is difficult to obtain, we excluded watermelon from fruit intake levels in subsequent analyses. Total fruit intake was not associated with risk of endometrial cancer. There was no association between risk of endometrial cancer and intake of individual fruits, including citrus fruits, apples, watermelon, grapes, bananas, peaches, pears, and all other fruits (data not shown).

The association of consumption of certain fruits and vegetables and endometrial cancer was further evaluated stratifying by menopausal status (Table 3). Total vegetable and dark green/dark yellow vegetable consumption were inversely associated with endometrial cancer risk for both pre- and postmenopausal women, although the point estimates and trend test did not reach statistical significance. Allium vegetable intake was associated with a significantly reduced risk of endometrial cancer in premenopausal women, with an adjusted OR for the highest quartile being 0.41 (95% CI=0.24–0.71). Consumption of fresh legumes, on the other hand, was inversely associated with endometrial cancer risk (OR=0.58, 95% CI=0.39–0.87 for the highest quartile) among postmenopausal women.

Table 4 shows results of additional analyses stratified by BMI. Among women with a BMI<25, increased consumption of total vegetables, and dark green/dark yellow vegetables, was inversely related to the risk of endometrial cancer (*P* for trend⩽0.05). The ORs and 95% CI for the highest *vs* lowest quartiles of intake of these vegetables were 0.61 (0.40–0.93), and 0.66 (0.44–0.97), respectively. Allium vegetable intake was found to be associated with a marginally significant reduction in endometrial cancer risk among thinner women (OR=0.70, 95% CI=0.47–1.06, for the highest quartile). Among overweight women (BMI>25), the inverse association between total vegetable and dark green/dark yellow vegetable intakes and endometrial cancer risk was less evident and not statistically significant. No significant interaction between vegetable intake and BMI or menopausal status was observed. Additional adjustment for soyfood consumption did not appreciably change the risk estimates presented in Tables 2, 3 and 4.

## DISCUSSION

This large, population-based, case–control study revealed an inverse relationship of endometrial cancer risk with increased consumption of total vegetables, dark green/dark yellow vegetables, fresh legumes, and allium vegetables. This association was independent of other major risk factors, including total energy intake, meat and fish intake. We found no association between fruit consumption and risk of endometrial cancer.

High consumption of fruits and vegetables has been associated with a reduced risk for many cancers such as cancer of the oral cavity, oesophagus, lung, stomach, and colorectum (Rao, 1996; Riboli and Norat, 2003; Key *et al*, 2004). Previous studies of endometrial cancer and fruits and vegetables, however, had limited and mixed results. Some case–control studies have reported an inverse association of endometrial cancer and higher consumption of certain vegetables and fruits (La Vecchia *et al*, 1986; Barbone *et al*, 1993; Levi *et al*, 1993; Goodman *et al*, 1997a, 1997b; McCann *et al*, 2000; Key *et al*, 2004); whereas several others did not (Shu *et al*, 1993; Petridou *et al*, 2002; Terry *et al*, 2002). Results from two cohort studies failed to find an association for either plant-based food groups or for micronutrients derived from fruits and vegetables (Zheng *et al*, 1995; Jain *et al*, 2000). In our study, the inverse association of vegetable intake and endometrial cancer was predominantly observed for dark green and dark yellow vegetables, including bok choy, spinach, fresh green peppers, garlic shoots, chives, Chinese celery, scallions, carrots, and sweet potatoes. Many of these are not commonly consumed by women in Western countries, so the lack of association found by earlier studies may reflect this low consumption and/or small variations in consumption. Our findings are consistent with other epidemiological studies linking reduced risk for hormone-related cancers with dark green/dark yellow vegetables consumption (McCann *et al*, 2000; Cramer *et al*, 2001; Littman *et al*, 2001; Malin *et al*, 2003). Dark green/dark yellow vegetables contain high levels of carotenoids, folates, vitamin C, and riboflavin. Carotenoids and vitamin C may inhibit endometrial carcinogensis via antioxidant effects, while folate influences DNA stability via its important role in the synthesis of nucleotides and DNA methylation. Folate also could affect carcinogenesis in numerous specific cancers (Lucock 2000; Kim 2000). However, we did not find fruit consumption to be related to a lower risk of endometrial cancer, which is consistent with findings from some previous studies (Shu *et al*, 1993; Petridou *et al*, 2002; Terry *et al*, 2002), but disagrees with others (Levi *et al*, 1993; Goodman *et al*, 1997a, 1997b; Littman *et al*, 2001). Differences in study design and consumption levels across studies may have contributed to the controversy. We found that higher intake of fresh legumes is inversely associated with endometrial cancer risk, especially among postmenopausal women. Fresh soybeans are the main contributor to this group of foods, accounting for 42% of fresh legumes. Soybeans are rich source of phyto-oestrogens, which compete with oestrogen to bind oestrogen receptors and affect endogenous oestrogen synthesis, bioavailability, and clearance. Our results are consistent with our earlier report of total soyfood consumption and other studies on soyfood intake and endometrial cancer (Goodman *et al*, 1997a, 1997b; Horn-Ross *et al*, 2003; Xu *et al*, 2004).

Allium vegetables have previously been associated with a decreased risk of cancer of the stomach, intestine, bladder, and prostate (Bianchini and Vainio 2001; Hsing *et al*, 2002). The anticancer effect of allium vegetables may be attributed primarily to organosulphur compounds (OSCs), including diallyl sulphide, diallyl disulphide, and diallyl trisulphide (Herman-Antosiewiza and Singh 2004). To our knowledge, the association of allium vegetable consumption and endometrial cancer risk has only previously been examined in a smaller study (268 cases, 268 controls) that we conducted in Shanghai with a nonsignificant inverse association Shu *et al*, 1993). In the current study, the reduced risk of endometrial cancer associated with allium vegetable consumption was restricted to premenopausal women and persisted after adjustment for multiple known risk and protective factors for endometrial cancer including soyfood intake. More studies are needed to further evaluate the effect of allium vegetables on endometrial cancer and the possible underlying biological mechanisms involved.

Strengths of our study include the population-based study design, large sample size, and detailed information on a wide range of potential confounders. Additionally, the FFQ used in the study has been validated and covers most usual foods in Shanghai. However, several limitations, common to other observational studies on diet and cancer, should be mentioned. Due to the nature of self-reported dietary information, misclassification of fruit and vegetable consumption is unavoidable, which may bias the diet and endometrial cancer association. While nondifferential misclassification is likely to bias the result towards the null, differential misclassification can be biased in either direction and should be taken into consideration. In order to minimise the potential recall bias attributed to dietary change related to cancer diagnosis, all cases were asked about their diet during the 5 years prior to cancer diagnosis. The median (25–75th percentile) interval between diagnosis and interview for cases was 5 (3–8) months, which minimised the influence of disease related to dietary change on recall of usual dietary habits prior to disease diagnosis. As part of the study, we also collected information on whether study participants had changed their diet in the week before the interview. Approximately, 10% of cases and 1% of controls reported significant dietary changes. Further analyses restricted to subjects reporting no dietary change showed no substantial change in the observed vegetable intake and endometrial cancer association. With 15.3% of cases and 27.4% of controls refusing to participate in the study, selection bias could be a concern. However, the median intake of fruits and vegetables in this study was similar to those found in a previous study of breast cancer that we conducted in Shanghai using the same FFQ, which had a much higher (92%) response rate.

In summary, this population-based, case–control study of the risk of endometrial cancer found an inverse association between vegetables, particularly dark green/dark yellow vegetables, legumes, and allium vegetables, with endometrial cancer risk. Further studies are needed to investigate the biochemicals responsible for the inverse association and to clarify the biological mechanisms involved.

## Figures and Tables

**Table 1:** Comparison of cases and controls on demographics and selected endometrial cancer risk factors^a^

	**Cases (*n*=832)**	**Controls (*n*=846)**	***P*-value**
Age (mean±s.d.)	54.8±8.60	55.2±8.58	0.33
			
*Education (%)*			
No formal education	9.0	12.8	
Elementary education	15.5	14.9	
Middle and high school	60.5	60.6	
Professional, college, and above	15.0	11.7	0.03
			
*Marital status (%)*			
Unmarried	1.7	1.2	
Married or cohabiting	87.0	87.7	
Separated/divorced/bereft of spouse	11.3	11.1	0.68
			
*Per capita income in previous year (yuan) (%)*			
⩽4166.7	27.6	28.8	
4166.8–6250.3	29.3	28.7	
6250.4–8333.3	6.9	5.9	
⩾8333.3	36.2	36.5	0.83
			
*Number of pregnancies (%)*			
Never	7.5	4.1	
1	16.5	12.9	
2	23.9	24.6	
3	23.3	24.5	
4	17.0	18.6	
⩾5	11.9	15.4	<0.01
			
Cancer among first-degree relative (%)^b^	8.1	3.1	<0.01
Natural menopause (%)	57.7	62.7	0.03
Regular alcohol consumption (%)	2.4	5.0	<0.01
Hormone replace therapy (HRT) (%)	4.2	4.0	0.85
Oral contraceptive (OC) use (%)	17.7	24.5	<0.01
Regular exercise (%)	30.3	33.7	0.14
Age at menarche^c^	14 (13,16)	15 (13, 16)	<0.0001
Age at menopause (among postmenopausal women)^c^	50.1 (48.6, 52.5)	49.4 (47.1, 51.1)	<0.0001
Years of menstruation^c^	33.2 (29.9, 36.1)	31.4 (27.8, 34.4)	<0.0001
Body mass index (BMI)^c^	25.1 (22.7, 27.9)	23.7 (21.4, 26.3)	<0.0001
Waist-to-hip circumference ratio (WHR)^c^	0.84 (0.81, 0.87)	0.82 (0.78, 0.85)	<0.0001
Usual energy intake (kcal day^−1^)^c^	2171.2 (1871.4, 2497.0)	2141.5 (1840.8, 2485.2)	0.71
Total meat and fish intake (g day^−1^)^c^	115.2 (74.7, 173.7)	102.0 (63.7, 154.2)	<0.01

a Subjects with missing values were excluded from the analysis.

b Family history of colorectal, endometrial, and breast cancer among first-degree relatives

c Median (25th, 75th percentile) are presented.

**Table 2:** Association of endometrial cancer risk with consumption levels of fruits and vegetables

	**Cases/controls**	**OR (95% CI)†**
*Total vegetables (g day*^−1^)		
⩽179.22	207/212	1.00
179.23–278.68	219/211	0.95 (0.70–1.27)
278.69–404.15	210/212	0.86 (0.64–1.17)
>404.15	196/211	0.69 (0.50–0.96)
*P* for trend		0.02
		
*Dark green*^a^*/dark yellow vegetables*^b^ *(g day*^−1^)		
⩽54.78	225/211	1.00
54.79–93.13	218/212	0.90 (0.68–1.20)
93.14–146.19	205/211	0.84 (0.63–1.12)
>146.19	184/212	0.70 (0.52–0.95)
*P* for trend		0.02
		
*Cruciferous vegetables*^c^ *(g day*^−1^)		
⩽52.63	222/211	1.00
52.64–91.38	227/212	1.02 (0.77–1.36)
91.39–137.38	166/211	0.70 (0.52–0.94)
>137.38	217/212	0.83 (0.62–1.12)
*P* for trend		0.06
		
*Fresh legumes*^d^ *(g day*^−1^)		
⩽8.66	212/213	1.00
8.67–17.85	210/211	0.90 (0.67–1.21)
17.86–35.35	223/211	0.89 (0.66–1.20)
>35.35	187/211	0.70 (0.52–0.96)
*P* for trend		*P*=0.003
		
*Allium vegetables*^e^ *(g day*^−1^)		
⩽0.99	204/213	1.00
1.01–3.81	227/210	1.14 (0.85–1.52)
3.82–9.59	212/211	0.96 (0.71–1.29)
>9.59	189/212	0.76 (0.56–1.03)
*P* for trend		*P*=0.04
		
*Melons*^f^ *(g day*^−1^)		
⩽18.47	215/211	1.00
18.48–35.75	178/216	0.66 (0.49–0.89)
35.76–62.49	203/207	0.83 (0.62–1.13)
>62.49	236/212	0.83 (0.62–1.12)
*P* for trend		*P*=0.54
		
*Mushrooms* *(g day*^−1^)		
⩽0.67	187/205	1.00
0.68–2.03	180/183	0.96 (0.70–1.31)
2.04–6.79	239/262	0.83 (0.62–1.11)
>6.79	226/196	0.98 (0.72–1.34)
*P* for trend		0.67
		
*Tomatoes (g day*^−1^)		
⩽8.65	186/184	1.00
8.66–23.09	255/252	1.02 (0.76–1.37)
23.10–46.18	151/183	0.76 (0.55–1.05)
>46.18	240/227	0.95 (0.70–1.29)
*P* for trend		0.42
		
*Fruits*^g^ *(total without watermelon) (g day*^−1^)		
⩽39.03	213/211	1.00
39.04–83.54	189/212	0.96 (0.71–1.30)
83.55–143.43	228/211	1.20 (0.89–1.61)
>143.43	202/212	0.97 (0.72–1.31)
*P* for trend		0.79

† Adjusted for age, education, menopausal status, years of menstruation, first-degree family history of breast, colorectal, endometrial cancer, OC use, number of pregnancies, history of diabetes, BMI, total meat and fish intake and caloric intake.

a Dark green vegetables: bok choy, spinach, fresh green peppers, garlic shoots, chives, scallions, Chinese celery.

b Dark yellow vegetables: carrots, sweet potato.

c Cruciferous vegetables: bok choy, cabbage, napa cabbage, cauliflower, Chinese white turnip.

d Fresh legumes: fresh soybean, fresh broad beans, yard long beans, green beans, hyacinth beans/snow peas.

e Allium vegetables: garlic, head of garlic, chives, scallions, garlic shoots.

f Melons: water melons, cucumbers, wax gourds.

g Fruits: apples, peaches, pears, grapes, tangerines, oranges, grapefruits, bananas, strawberries, and cantaloupe.

**Table 3:** Association of endometrial cancer risk with consumption levels of selected vegetables by menopausal status

	**Q1**	**Q2**	**Q3**	**Q4**	***P* for trend**
*Premenopausal* a					
Total vegetables	1.00	0.75 (0.46–1.24)	0.84 (0.52–1.37)	0.59 (0.35–1.02)	0.10
Dark green/dark yellow vegetables	1.00	0.71 (0.44–1.15)	0.62 (0.38–1.01)	0.64 (0.39–1.06)	0.07
Cruciferous vegetables	1.00	0.97 (0.60–1.56)	0.55 (0.33–0.92)	0.95 (0.58–1.54)	0.44
Fresh legumes	1.00	0.79 (0.47–1.32)	0.97 (0.59–1.59)	1.12 (0.68–1.84)	0.78
Allium vegetables	1.00	1.25 (0.78–2.01)	0.95 (0.59–1.55)	0.41 (0.24–0.71)	<0.01
Melons	1.00	0.83 (0.50–1.36)	0.77 (0.46–1.28)	1.09 (0.68–1.77)	0.70
Tomatoes	1.00	0.97 (0.59–1.57)	0.70 (0.42–1.14)	0.84 (0.52–1.35)	0.29
Total fruits (without watermelon)	1.00	1.04 (0.64–1.70)	1.07 (0.66–1.74)	0.88 (0.83–1.44)	0.64
					
*Postmenopausal* a					
Total vegetables	1.00	1.19 (0.82–1.72)	0.85 (0.58–1.26)	0.76 (0.51–1.14)	0.07
Dark green/dark yellow vegetables	1.00	1.06 (0.73–1.53)	1.02 (0.70–1.47)	0.74 (0.50–1.09)	0.14
Cruciferous vegetables	1.00	1.20 (0.83–1.73)	0.92 (0.63–1.35)	0.93 (0.64–1.37)	0.42
Fresh legumes	1.00	1.05 (0.73–1.52)	0.83 (0.57–1.21)	0.58 (0.39–0.87)	<0.01
Allium vegetables	1.00	1.10 (0.76–1.61)	0.92 (0.63–1.34)	1.00 (0.69–1.47)	0.80
Melons	1.00	0.63 (0.43–0.93)	0.87 (0.60–1.27)	0.78 (0.53–1.15)	0.53
Tomatoes	1.00	1.02 (0.70–1.47)	0.79 (0.52–1.19)	0.95 (0.64–1.39)	0.53
Total fruits (without watermelon)	1.00	0.77 (0.52–1.14)	1.07 (0.73–1.57)	0.90 (0.61–1.43)	0.96

a Adjusted for age, education, years of menstruation, first-degree family history of breast, colorectal, and endometrial cancer, history of diabetes, OC use, number of pregnancies, BMI, total meat and fish intake, and total caloric intake.

**Table 4:** Association of endometrial cancer risk with consumption levels of selected vegetables by BMI status

	**Q1**	**Q2**	**Q3**	**Q4**	***P* for trend**
BMI <25^a^					
Total vegetables	1.00	0.81 (0.55–1.19)	0.89 (0.60–1.31)	0.61 (0.40–0.93)	0.04
Dark green/dark yellow vegetables	1.00	0.78 (0.53–1.14)	0.68 (0.46–1.00)	0.66 (0.44–0.97)	0.03
Cruciferous vegetables	1.00	1.07 (0.73–1.57)	0.82 (0.56–1.21)	0.87 (0.59–1.30)	0.29
Fresh legumes	1.00	0.90 (0.61–1.32)	0.81 (0.55–1.20)	0.79 (0.53–1.18)	0.21
Allium vegetables	1.00	1.30 (0.89–1.89)	1.14 (0.77–1.66)	0.70 (0.47–1.06)	0.09
Melons	1.00	0.63 (0.42–0.95)	0.81 (0.54–1.19)	0.76 (0.51–1.13)	0.40
Tomatoes	1.00	1.11 (0.76–1.62)	0.69 (0.45–1.06)	0.88 (0.89–1.33)	0.18
Total fruits (without watermelon)	1.00	0.74 (0.50–1.10)	1.02 (0.70–1.50)	0.77 (0.52–1.15)	0.49
					
BMI⩾25^a^					
Total vegetables	1.00	1.16 (0.74–1.81)	0.98 (0.62–1.56)	0.88 (0.54–1.43)	0.46
Dark green/dark yellow vegetables	1.00	1.10 (0.70–1.72)	1.12 (0.72–1.76)	0.82 (0.52–1.29)	0.42
Cruciferous vegetables	1.00	1.03 (0.67–1.60)	0.68 (0.43–1.09)	0.96 (0.62–1.49)	0.50
Fresh legumes	1.00	0.91 (0.58–1.44)	1.09 (0.70–1.70)	0.62 (0.38–1.00)	0.13
Allium vegetables	1.00	0.92 (0.59–1.43)	1.16 (0.74–1.82)	0.74 (0.46–1.18)	0.41
Melons	1.00	0.83 (0.53–1.29)	0.68 (0.42–1.09)	1.03 (0.65–1.62)	0.96
Tomatoes	1.00	0.89 (0.57–1.39)	0.88 (0.54–1.43)	1.07 (0.68–1.67)	0.72
Total fruits (without watermelon)	1.00	1.41 (0.90–2.23)	1.44 (0.91–2.28)	1.34 (0.84–2.12)	0.26

a Adjusted for age, education, years of menstruation, menopausal status, first-degree family history of breast, colorectal, and endometrial cancer, history of diabetes, OC use, number of pregnancies, total meat and fish intake, and total caloric intake.

## References

[bib1] Barbone F, Austin H, Partridge EE (1993) Diet and endometrial cancer: a case–control study. Am J Epidemiol 137: 393–403 8460621 10.1093/oxfordjournals.aje.a116687

[bib2] Bianchini F, Vainio H (2001) Allium vegetables and organosulfur compounds: do they help prevent cancer? Environ Health Perspect 109: 893–902 11673117 10.1289/ehp.01109893 PMC1240438

[bib3] Cramer DW, Kuper H, Harlow BL, Titus-Ernstoff L (2001) Carotenoids, antioxidants and ovarian cancer risk in pre- and postmenopausal women. Int J Cancer 94: 128–134 11668487 10.1002/ijc.1435

[bib4] Goodman MT, Hankin JH, Wilkens LR, Lyu LC, McDuffie K, Liu LQ, Kolonel LN (1997a) Diet, body size, physical activity, and the risk of endometrial cancer. Cancer Res 57: 5077–5085 9371506

[bib5] Goodman MT, Wilkens LR, Hankin JH, Lyu LC, Wu AH, Kolonel LN (1997b) Association of soy and fiber consumption with the risk of endometrial cancer. Am J Epidemiol 146: 294–306 9270408 10.1093/oxfordjournals.aje.a009270

[bib6] Herman-Antosiewiza A, Singh SV (2004) Signal transduction pathways leading to cell cycle arrest and apoptosis induction in cancer cells by allium vegetable-derived organosulfur compounds: a review. Mutat Res 555: 121–131 15476856 10.1016/j.mrfmmm.2004.04.016

[bib7] Horn-Ross PL, John EM, Canchola AJ, Stewart SL (2003) Phytoestrogen intake and endometrial cancer risk. J Natl Cancer Inst 95: 1158–1164 12902445 10.1093/jnci/djg015

[bib8] Hsing AW, Chokkalingam AP, Gao YT, Madigan MP, Deng J, Gridley G, Fraumeni Jr JF (2002) Allium vegetables and risk of prostate cancer: a population-based study. J Natl Cancer Inst 94: 1648–1651 12419792 10.1093/jnci/94.21.1648

[bib9] Jain MG, Rohan TE, Howe GR, Miller AB (2000) A cohort study of nutritional factors and endometrial cancer. Eur J Epidemiol 16: 899–905 11338120 10.1023/a:1011012621990

[bib10] Key TJ, Schatzkin A, Willett WC, Allen NE, Spencer EA, Travis RC (2004) Diet, nutrition and the prevention of cancer. Public Health Nutr 7: 187–200 14972060 10.1079/phn2003588

[bib11] Kim Y-I (2000) Methylenetetrahydrofolate reductase polymorphisms, folate, and cancer risk: a paradigm of gene–nutrient interactions in carcinogenesis. Nutr Rev 58: 205–209 10941256 10.1111/j.1753-4887.2000.tb01863.x

[bib12] La Vecchia C, Decarli A, Fasoli M, Gentile A (1986) Nutrition and diet in the etiology of endometrial cancer. Cancer 57: 1248–1253 3002600 10.1002/1097-0142(19860315)57:6<1248::aid-cncr2820570631>3.0.co;2-v

[bib13] Levi F, Franceschi S, Negri E, La Vecchia C (1993) Dietary factors and the risk of endometrial cancer. Cancer 71: 3575–3581 8490907 10.1002/1097-0142(19930601)71:11<3575::aid-cncr2820711119>3.0.co;2-0

[bib14] Littman AJ, Beresford SAA, White E (2001) The association of dietary fat and plant foods with endometrial cancer (United States). Cancer Causes Control 12: 691–702 11562109 10.1023/a:1011292003586

[bib15] Lucock M (2000) Folic acid: nutritional biochemistry, molecular biology, and role in disease processes. Mol Genet Metab 71: 121–138 11001804 10.1006/mgme.2000.3027

[bib16] Malin AS, Qi D, Shu XO, Gao YT, Friedmann JM, Jin F, Zheng W (2003) Intake of fruits, vegetables and selected micronutrients in relation to the risk of breast cancer. Int J Cancer 105: 413–418 12704679 10.1002/ijc.11088 PMC1780272

[bib17] McCann SE, Freudenheim JL, Marshall JR, Brasure JR (2000) Diet in the epidemiology of endometrial cancer in Western New York (United States). Cancer Causes Control 11: 965–974 11142531 10.1023/a:1026551309873

[bib18] Parkin DM, Whelan SL, Ferlay J, Teppo L, Thomas DB (2003) Cancer Incidence in Five Continents Vol VIII. Lyon, France: International Agency for Research on Cancer (IARC) press

[bib19] Petridou E, Kedikoglou S, Koukoulomatis P, Dessypris N, Trichopoulos D (2002) Diet in relation to endometrial cancer risk: a case–control study in Greece. Nutr Cancer 44: 16–22 12672637 10.1207/S15327914NC441_3

[bib20] Purdie DM, Green AC (2001) Epidemiology of endometrial cancer. Best Pract Res Clin Obstet Gynaecol 15: 341–354 11476557 10.1053/beog.2000.0180

[bib21] Rao GN (1996) Influence of diet on tumors of hormonal tissues. Prog Clin Biol Res 394: 41–56 8778807

[bib22] Riboli E, Norat T (2003) Epidemiologic evidence of the protective effect of fruit and vegetables on cancer risk. Am J Clin Nutr 78: 559S–569S 12936950 10.1093/ajcn/78.3.559S

[bib23] Shu XO, Zheng W, Potischman N, Brinton LA, Hatch MC, Gao YT, Fraumeni Jr JF (1993) A population-based case–control study of dietary factors and endometrial cancer in Shanghai, People's Republic of China. Am J Epidemiol 137: 155–165 8452119 10.1093/oxfordjournals.aje.a116655

[bib24] Terry P, Vainio H, Wolk A, Weiderpass E (2002) Dietary factors in relation to endometrial cancer: a national case–control study in Sweden. Nutr Cancer 42: 25–32 12235647 10.1207/S15327914NC421_4

[bib25] Xu WH, Zheng W, Xiang YB, Ruan ZX, Cheng JR, Dai Q, Gao YT, Shu XO (2004) Soya food intake and risk of endometrial cancer among Chinese women in Shanghai: population based case–control study. BMJ 328: 1285 15136343 10.1136/bmj.38093.646215.AE PMC420166

[bib26] Zheng W, Kushi LH, Potter JD, Sellers TA, Doyle TJ, Bostick RM, Folsom AR (1995) Dietary intake of energy and animal foods and endometrial cancer incidence. The Iowa women's health study. Am J Epidemiol 142: 388–394 7625403 10.1093/oxfordjournals.aje.a117646

